# Accuracy of EEG Slow Wave Activity in Predicting Favorable Outcome in Patients With Hypoxic Brain Injury—A Protocol for a Substudy of the STEPCARE Trial

**DOI:** 10.1111/aas.70126

**Published:** 2025-09-17

**Authors:** Johanna Hästbacka, Erik Westhall, Marion Moseby‐Knappe, Marjaana Tiainen, Anna Lybeck, Matti Reinikainen, Helena Levin, Markus B. Skrifvars, Joonas Tirkkonen, Janus C. Jakobsen, Niklas Nielsen, Jussi Toppila, Marjolein M. Admiraal, Jukka Kortelainen

**Affiliations:** ^1^ Department of Intensive Care, Faculty of Medicine and Health Technology Tampere University Hospital, Wellbeing Services County of Pirkanmaa and Tampere University Tampere Finland; ^2^ Department of Clinical Sciences Lund Lund University Lund Sweden; ^3^ Department of Clinical Neurophysiology Skåne University Hospital Lund Sweden; ^4^ Department of Neurology and Rehabilitation Skåne University Hospital Lund Sweden; ^5^ Department of Neurology Helsinki University Hospital and University of Helsinki Helsinki Finland; ^6^ Department of Anaesthesiology and Intensive Care Kuopio University Hospital, University of Eastern Finland Kuopio Finland; ^7^ Department of Research, Development, Education and Innovation Skåne University Hospital Lund Sweden; ^8^ Department of Anaesthesia and Intensive Care Medicine Helsinki University Hospital and University of Helsinki Helsinki Finland; ^9^ Copenhagen Trial Unit, Centre for Clinical Intervention Research, Capital Region of Denmark, Rigshospitalet, Copenhagen University Hospital Copenhagen Denmark; ^10^ Department of Regional Health Research, the Faculty of Health Sciences University of Southern Denmark Sønderborg Denmark; ^11^ Department of Anaesthesia and Intensive Care Helsingborg Hospital Helsingborg Sweden; ^12^ Department of Clinical Neurophysiology HUS Diagnostic Center, Helsinki University Hospital and University of Helsinki Helsinki Finland; ^13^ Center for Machine Vision and Signal Analysis University of Oulu Oulu Finland

## Abstract

**Background:**

Early restitution of electroencephalogram (EEG) slow wave activity (SWA) may be able to predict favorable functional outcome after cardiac arrest. SWA can be monitored using the C‐Trend Index, a recently developed dynamic numerical index computed using commercially available medical device software.

**Methods:**

This is a pre‐planned prospective substudy of the randomized controlled Sedation, TEmperature and Pressure after Cardiac Arrest and REsuscitation (STEPCARE) trial. With a factorial design, the STEPCARE trial evaluates the effects of three different interventions (sedation, temperature management, and mean arterial blood pressure) on functional outcomes in adult out‐of‐hospital cardiac arrest (OHCA) patients. We will record continuous EEG (cEEG) starting as early as possible after ICU admission. We will compare the accuracy (the proportion of correct predictions of all predictions) of C‐Trend Index with that of blinded retrospective visual analysis of cEEG at 12 h after return of spontaneous circulation in predicting favorable functional outcome (modified Rankin Scale 0–3) 6 months after cardiac arrest. We aim to recruit 300 patients to show noninferiority in prognostic accuracy of the C‐Trend Index compared with the visual analysis of cEEG, using a 2% limit for noninferiority. Furthermore, we will assess whether the therapeutic intervention related to sedation, carried out as a part of the STEPCARE trial modifies the performance of the EEG‐based predictors.

**Conclusion:**

The study will compare the accuracy of SWA measured using C‐Trend Index with the gold standard, visual analysis of cEEG, in predicting favorable functional outcome after OHCA. The study will also assess the effect of the sedation intervention of the STEPCARE trial on the predictive accuracy of C‐Trend Index. If the accuracy of the C‐Trend Index is non‐inferior to the comparator's, it may provide a feasible and easy‐to‐learn bedside method, especially in hospitals with limited availability of neurophysiology expertise.

**Trial Registration:**

ClinicalTrials.gov identifier: NCT06564675

## 1 Background and Significance

Guidelines recommend electroencephalography (EEG) assessment after cardiac arrest as a part of the multimodal neuroprognostication of functional outcome [1, 2]. The application of EEG in post‐resuscitation care is often limited by the restricted availability of experts with adequate training to initiate recordings and interpret results during office hours [3].

Traditionally, recommendations have mainly focused on prognosticating unfavorable outcome after cardiac arrest, but recently predicting favorable outcomes has gained attention in recommendations [4]. Early indicators of favorable outcome deserve more attention as they might support, for example, shortening of the sedation after cardiac arrest potentially leading to earlier discharge from the intensive care unit.

Several specific EEG features, such as early return of continuous and normal‐voltage background activity, are often indicative of a favorable outcome, and may thus provide valuable prognostic information already during the first 24 h in the intensive care unit (ICU) [5, 6, 7, 8, 9]. In a Finnish two‐centre study, we recently found that EEG slow wave activity (SWA) during the first 24 h is a promising measure for outcome prediction after cardiac arrest in patients sedated with propofol [10]. In that study, we used C‐Trend Index, a parameter provided by a commercially available medical device software, as a measure of SWA. SWA is a normal feature in EEG during physiological sleep and medical sedation, which supports the assumption that it is associated with normal brain function and favorable outcome after cardiac arrest [11].

Patients with unfavorable functional outcomes had significantly lower SWA during the early phase continuous EEG monitoring (cEEG) compared to those with a favorable functional outcome. A C‐Trend Index below a cutoff value of 20 had the best accuracy for predicting unfavorable outcome before 12 h after cardiac arrest, with a specificity and sensitivity of 95% (83%–100%, 95% Confidence interval) and 83% (70%–92%), respectively. The recordings were performed using a disposable, self‐adhesive frontal electrode and a wireless recording device, eliminating the need for qualified EEG technicians or specially trained nurses [10].

Here, we present the design and rationale behind the PROPEA STEPCARE, which will compare the ability of the C‐Trend Index to predict favorable functional outcomes at 6 months after cardiac arrest with the gold standard, visual analysis of EEG.

Previous studies indicate that improvement of the C‐Trend‐Index beyond a certain cutoff value or improvement of cEEG pattern to a nearly continuous normal‐voltage background at an early time point after cardiac arrest predicts favorable outcome [10, 11, 12, 13].

Our primary objective is to compare the early predictive accuracy of C‐Trend Index cutoff value 20 with blinded retrospective visual analysis of cEEG in predicting favorable functional outcome at 6 months after cardiac arrest. Furthermore, we will assess the effect of the depth of sedation on the predictive ability of cEEG and C‐Trend Index. Comparing minimal sedation with deep sedation is one of the three interventions carried out as part of the STEPCARE trial [14]. The effect of sedation on the predictive ability of cEEG has been identified as a knowledge gap in the recent international consensus recommendation [4].

We hypothesize that the accuracy of the C‐Trend Index is non‐inferior in predicting favorable outcomes at 12 h after return of spontaneous circulation (ROSC) compared to a retrospective visual analysis of cEEG, blinded to the studied therapeutic intervention and outcome, and that the accuracy will not be impaired by the sedation intervention.

## 2 Methods

We will report the study according to the Standards of Reporting of Diagnostic Accuracy Studies (STARD) guidelines [15]. The PROPEA STEPCARE is a prospective observational substudy of the Sedation, Temperature and Pressure after Cardiac Arrest and Resuscitation (STEPCARE) trial, a prospective randomized multi‐centre trial with a factorial design. STEPCARE assesses the effects of three different interventions (minimal or deep sedation, temperature management with a device targeting ≤ 37.5°C or conservative management without a device, target level of MAP > 65 or > 85 mmHg) on mortality and functional outcome of adult patients with an out‐of‐hospital cardiac arrest (OHCA) with a non‐traumatic cause [14, 16, 17, 18].

### 2.1 Patient Population

The study population is comprised of patients participating in the STEPCARE trial. The study will be conducted in 5–8 selected centres with access to equipment for registering cEEG using the BrainStatus device (Bittium, Oulu, Finland). We will include adult OHCA patients with sustained ROSC, who remain comatose after resuscitation (no response to verbal commands or Full Outline of UnResponsiveness score motor response < 4), and who have no limitations of care. Patients with trauma or hemorrhage as the reason for arrest, patients with suspected or confirmed intracranial hemorrhage, pregnant patients, and those previously randomized to STEPCARE will be excluded [14, 16, 17, 18]. Patients with known allergy to adhesive material and/or injured skin in the frontal‐temporal area preventing the use of the adhesive electrode will be excluded.

### 2.2 Outcomes

Functional outcome will be assessed at 6 months follow‐up by an investigator blinded to EEG data and trial interventions [14, 16, 17, 18]. Favorable outcome will be defined as modified Rankin Scale (mRS) 0–3 and unfavorable outcome as modified Rankin Scale 4–6.

### 2.3 Ethics

The consent for the STEPCARE trial includes EEG assessment in the multimodal prognostication, regardless of the type of recording. The protocol of the STEPCARE trial, including a deferred consent policy, has been approved by the ethics board of Helsinki University Hospital (HUS/17543/2022) and all other participating sites. All patients are treated according to the Declaration of Helsinki and its later amendments. The Finnish Medical Authority waived the need for a specific permission regarding device research.

### 2.4 Primary Study Question

Is the accuracy of the C‐Trend Index non‐inferior to the blinded retrospective visual analysis of cEEG in predicting favorable outcomes of cardiac arrest patients at 12 h after ROSC?

### 2.5 Secondary Study Question

Is the accuracy of C‐Trend Index and visual analysis of cEEG in predicting favorable outcome affected by the sedation intervention of the STEPCARE trial?

### 2.6 Other Study Questions

What is the prognostic value of C‐Trend Index in terms of sensitivity and specificity when using predefined cut‐off values above 50 and 80 in predicting favorable outcome?

What is the prognostic value of C‐Trend Index in terms of sensitivity and specificity when using a predefined cut‐off value below 20 to predict unfavorable outcome?

### 2.7 Management and Study Procedures

The management in the ICU is according to the STEPCARE trial protocol [14, 16, 17, 18]. A cEEG recording on randomized patients will be initiated as soon as possible after admission to the ICU and continued at least 24 h from ROSC or until the patient awakens to obey verbal commands, whichever comes first. The recording is carried out with a disposable self‐adhesive BrainStatus electrode (Bittium Oyj, Oulu, Finland) attached to the forehead of the patient, including 10 EEG electrodes on positions Fp1, Fp2, Af7, Af8, F7, F8, Sp1, Sp2, T9, and T10, and a wireless BrainStatus recording device (Bittium Oyj, Oulu, Finland). The treating clinical team can decide whether to add more EEG electrodes in addition to the BrainStatus electrode when monitoring cEEG or the performance of intermittent routine EEGs. Electrode placement and initiation of recording are performed according to written instructions.

### 2.8 Data Extraction

Original raw EEG signals are displayed on a bedside monitor at sites that use cEEG for routine monitoring of cardiac arrest patients and may be used as part of the multimodal prognostication defined in the STEPCARE protocol. The cEEG data are stored on a bedside computer or hospital server during the recording. The cEEG data are then pseudonymized before blinded retrospective analysis (EW, MA, JT). Amplitude‐integrated EEG and basic spectrogram analysis can be used to aid the retrospective visual analysis, but no other computational methods will be used. All cEEGs will be assessed by two out of three raters, according to the ACNS 2021 criteria [19], and inconsistencies between raters will be resolved by the third rater. The best EEG background pattern, present for at least five consecutive minutes, will be obtained in the hour between 11 and 12 h after CA. If recording quality is insufficient for visual analysis, the hour with the best recording quality closest to 12 h (12 h ± 3 h) after CA is used. At least 1 h of EEG recording with sufficient recording quality is required to be included in the analysis. cEEG analysis is performed by investigators blinded to the clinical outcome data and intervention group allocation after the recording of the last study participant has been finished. To minimize the risk of uncovering the allocated intervention, the cEEG recordings are accessible only for up to 15 h to the investigators performing the analysis. In all centres, including those using visible cEEG monitoring data for clinical purposes, treatment decisions on withdrawal of life support are based on ERC/ESICM guidelines [1], and strictly determined by the STEPCARE protocol [14, 16, 17, 18].

The C‐Trend Index values are calculated offline after the recording. The parameter is provided by C‐Trend (Cerenion, Oulu, Finland), a CE‐marked medical device software that utilizes artificial intelligence to capture several power and connectivity features in producing the C‐Trend Index. The parameter ranges from 0 to 100 in values, with higher values referring to higher normal SWA seen during propofol sedation. The parameter is not displayed on the bedside monitor and is not used during the clinical review of the cEEG data. Thus, all clinical and research personnel are blinded to the C‐Trend Index. A running 1‐h average value of the C‐Trend Index is automatically calculated, and for the analysis, the value is picked for each patient from the same time location used in the visual analysis of cEEG.

### 2.9 Statistical Analysis Plan and Data Analysis

Descriptive statistics with categorical values will be presented as numbers and percentages and continuous values with means with standard deviations (SD) or medians with interquartile ranges (IQR).

The sample size is calculated with regard to the primary outcome, that is the comparison of the prognostic accuracy of the C‐Trend Index and the visual assessment of cEEG at 12 h from ROSC as a reference standard. Firstly, we chose the expected sensitivity and specificity based on the study by Admiraal et al. [12], from which Sandroni et al. [13] calculated sensitivity (63.2%) and specificity (82.1%) for a favorable outcome for EEG with continuous or nearly continuous background with normal voltage, at 12 h from ROSC. Expecting a favorable outcome prevalence of 50%, the accuracy of 72.7% was calculated. The estimated accuracy of the C‐Trend Index used for the calculation was 86.08%, based on our previous work [10]. Accuracy stands for the number and percentage of correctly classified patients, calculated according to a formula (TP + TN)/(TP + TN + FP + FN), where TP and TN stand for the number of true positives and true negatives, respectively, and FP and FN stand for the number of false positives and false negatives, respectively [20]. Calculating the sample size for detecting non‐inferiority with a power of 0.90 and alpha of 0.05, and a non‐inferiority margin of 2%, a sample size of 230 patients is needed. To account for missing or poor‐quality recordings, and drop‐out, we aim to recruit 300 patients.

We will calculate the sensitivity, specificity, negative predictive value (NPV), positive predictive value (PPV), and positive (LR+) and negative (LR−) likelihood ratios with 95% CI for both methods. We will use McNemar's test to compare the two methods.

## 3 Results

The plan for presentation of the results is shown in the Supporting Information.

## 4 Discussion

We present the rationale and design of a prospective observational substudy of the international cardiac arrest 2 × 2 × 2 platform trial, STEPCARE, which includes adult patients with out‐of‐hospital cardiac arrest of aetiologies not limited to cardiac causes. We will analyse the predictive accuracy of the C‐Trend Index, a measure of the EEG SWA, and compare it with the gold standard, visual analysis of cEEG by investigators blinded to clinical data, in detecting favorable functional outcomes 6 months after OHCA. The C‐Trend Index is a dynamic numerical index provided as a commercially available medical device software. Based on a previous smaller study, accuracy non‐inferior to that of the gold standard is expected in the early phase after ROSC [10]. If this is confirmed, the dynamic numerical nature of the C‐Trend Index may become useful in the clinical care of OHCA patients, as the visual analysis of cEEG depends on specialized expertise in interpreting patterns. A numerical dynamic measure could be feasible for timely bedside interpretation by ICU personnel. Also, the monitoring setup with the frontal electrode and wireless recording device is rapid and easy to apply.

In this study we focus on predicting favorable functional outcome at an early phase after ROSC. The rationale is to provide early information to the patient's family and caregivers and to identify patients with potential for recovery despite eventual slow awakening or unstable clinical course. Furthermore, methods providing prognostic information early may be helpful in certain clinical decisions, such as the timing of for example, cardiac surgery and limiting the duration of sedation.

In the STEPCARE trial, 50% of the patients are randomized to minimal sedation, which allows a proportion of patients with good functional outcomes to wake up early, in some cases even before 12 h post‐arrest. Thus, some patients may not be included in the current study. However, we expect that most patients in the minimal sedation group and all patients in the deep sedation group will be unconscious at 12 h after ROSC. Further, awake patients obeying commands usually have a good functional outcome and thus do not require neuroprognostication. Although multimodal prognostication occurs in a later phase, an early prognostication may provide valuable information, especially for avoiding inadequate withdrawal of care in the future and encouraging continued care in patients with late awakening. As previous knowledge on the performance of the C‐Trend Index in outcome prediction is obtained from patients under sedation [10, 11], assessing the performance of the C‐Trend Index in patients allocated to minimal sedation is also important.

The strengths of this study include the prospective multicenter design with a blinded analysis of the cEEG, a comparison of the C‐Trend Index with a gold standard, and a large patient population with an a priori calculation of the sample size needed to detect at least a 10% difference in accuracy.

The limited number of participating centres in the STEPCARE trial, due to the special equipment required for cEEG recording, may influence the generalizability of the results.

In conclusion, we will compare the predictive accuracy of the C‐Trend numerical index based on EEG SWA with visual assessment of cEEG in predicting favorable functional outcome 6 months after OHCA. We will also assess the effect of the sedation intervention on the predictive accuracy of the C‐Trend Index. If the C‐Trend Index is shown to be non‐inferior in predicting favorable functional outcome, it may provide a valuable information for decision making for ICU clinical staff at an early phase in a dynamic numerical form.

## Author Contributions

Johanna Hästbacka drafted the manuscript. All other authors contributed to the study design and critically revised the manuscript. All authors approved the final version.

## Conflicts of Interest

Dr. Kortelainen is a co‐founder of Cerenion Oy (Finland).

## Supporting information


**Data S1:** Supporting Information.

## Data Availability

Data sharing not applicable to this article as no datasets were generated or analysed during the current study.
