# Supplementary material for: Accuracy of EEG Slow Wave Activity in Predicting Favorable Outcome in Patients With Hypoxic Brain Injury—A Protocol for a Substudy of the STEPCARE Trial
Source: Acta Anaesthesiol Scand. 2025 Sep 17;69(9):e70126. doi: 10.1111/aas.70126 (PMC12441756; doi:10.1111/aas.70126)
Supplement: Supplementary file 1 — Data S1: Supporting Information. [file AAS-69-0-s001.docx]

**Supplementary material to:**

Title: Accuracy of EEG Slow Wave Activity in Predicting Favourable Outcome in Patients with Hypoxic Brain Injury – A protocol for a substudy of the STEPCARE trial

Short title: PROPEA STEPCARE

Hästbacka J^1^, MD, Ph. D., (0000-0002-3613-7231),Westhall E^2, 3^, MD, Ph.D., Moseby-Knappe M^2, 4^, MD, Ph. D., Tiainen M^5^, MD, Ph. D., Lybeck A^2^, MD, Ph. D., Reinikainen M^6^, MD, Ph. D. (000-0001-6878-3740), Levin H^2,7^, MSc, Skrifvars MB^8^, MD, Ph.D.(0000 0002 0341 0262), Tirkkonen J^1^, MD, Ph. D., Jakobsen JC^9, 10^, MD, Ph. D., Nielsen N^2, 11^, MD, Ph. D., Toppila J^12^, MD, Ph.D., (0000-0001-9418-2519), Admiraal MM^2, 3^, MD, Ph. D., (0000-0002-3670-0370), Kortelainen J^13^, MD, Ph. D.

**Planned presentation of the results of the study.**

A flowchart of the patient population will be shown as an image.

**Table 1.** Baseline and clinical characteristics of participants

|  | Favourable outcome, N | Unfavourable outcome, N |
| --- | --- | --- |
| Age, years, median (IQR) |  |  |
| Sex, male, N (%) |  |  |
| Witnessed arrest, N (%) |  |  |
| Bystander CPR, N (%) |  |  |
| Bystander AED use, N (%) |  |  |
| First arrest rhythm shockable, N (%) |  |  |
| Time to ROSC, minutes, median (IQR) |  |  |
| STEMI, N (%) |  |  |
| Sedation  Minimal N (%)  Continuous deep N (%) |  |  |
| Temperature control  Without a feedback-controlled device, N (%)  With a feedback-controlled device, N (%) |  |  |
| MAP target  >65 mmHg, N (%)  >85 mmHg, N (%) |  |  |

**Table 2**. Comparison of sensitivity, specificity and accuracy of C-Trend Index and retrospective visual analysis of cEEG 12 hours after ROSC in predicting favourable functional outcomes in OHCA patients. The table also shows the sensitivity, specificity and accuracy of C-Trend Index according to the sedation intervention group and different cut-off values.

|  | True positives, N | True negatives, N | False negatives, N | False positives, N | Sensitivity % (95% CI) | Specificity % (95% CI) | Accuracy  (95% CI) |
| --- | --- | --- | --- | --- | --- | --- | --- |
| C-Trend Index 20 |  |  |  |  |  |  |  |
| Visual analysis of cEEG |  |  |  |  |  |  |  |
| p |  |  |  |  |  |  |  |
| **Minimal sedation** | | | | | | | |
| C-trend Index 20 |  |  |  |  |  |  |  |
| **Deep sedation** | | | | | | | |
| C-Trend Index 20 |  |  |  |  |  |  |  |
| **All patients** | | | | | | | |
| C-Trend Index 50 |  |  |  |  |  |  |  |
| C-Trend Index 80 |  |  |  |  |  |  |  |

**Table 3.** Performance of C-Trend Index at different cut-off values in predicting unfavourable outcome at 12 hours after ROSC.

|  | True positives, N | True negatives, N | False negatives, N | False positives, N | Sensitivity, % (95% CI) | Specificity, % (95% CI) | Accuracy  (95% CI) |
| --- | --- | --- | --- | --- | --- | --- | --- |
| C-Trend Index 20 |  |  |  |  |  |  |  |
| C-Trend Index 50 |  |  |  |  |  |  |  |
| C-Trend Index 80 |  |  |  |  |  |  |  |
